# Relevance of persistent perfusion deficits on clinical outcomes after successful endovascular treatment: a prospective serial magnetic resonance study

**DOI:** 10.3389/fneur.2025.1478240

**Published:** 2025-02-27

**Authors:** Adrián Valls Carbó, Alicia Palomar, Carlos Laredo, Mariano Werner, Laura Dorado, Sebastià Remollo, Josep Munuera, Josep Puig, Yolanda Silva, Natalia Pérez de la Ossa, Meritxell Gomis, Alejandro Bustamante, Carlos Castaño, Lucía Muñoz, Sira Domenech, Mikel Terceño, Mònica Millán, María Hernández-Pérez

**Affiliations:** ^1^Department of Neuroscience, Hospital Universitari Germans Trias i Pujol, Badalona, Spain; ^2^Canon Medical Systems Spain and Portugal, Barcelona, Spain; ^3^Institut d’Investigacions Biomèdiques Agustí Pi i Sunyer (IDIBAPS), Barcelona, Spain; ^4^Neuroradiology Service, Hospital Clínic de Barcelona, Barcelona, Spain; ^5^Department of Radiology, Hospital de la Santa Creu i Sant Pau, Barcelona, Spain; ^6^Department of Radiology (CDI) and IDIBAPS, Hospital Clínic Barcelona, Barcelona, Spain; ^7^Department of Neurology, Hospital Universitari de Girona Doctor Josep Trueta, Girona, Spain

**Keywords:** no-reflow, perfusion imaging, MRI, ischemic stroke, reperfusion

## Abstract

**Background:**

Half of the patients who undergo successful recanalization after endovascular treatment (EVT) experience poor clinical outcomes. Impaired microvascular reperfusion (IMR) may explain this lack of improvement, but its frequency and clinical significance remain unclear. The study aims to describe the frequency and associated factors of IMR.

**Materials and methods:**

We conducted a study on a cohort of patients with anterior large artery occlusion, treated with EVT at a single center, who achieved mTICI ≥2C. Perfusion MRI was obtained at arrival, up to 2 h after EVT (post-EVT MRI), and on day 5. IMR was observed only on the post-EVT relative cerebral blood volume (rCBV) maps as voxels within the follow-up ischemic lesion, exhibiting a > 15% asymmetry compared to a mirror homolog, in the absence of internal carotid occlusion, hemorrhagic transformation, or arterial reocclusion. Patients with an IMR volume greater than 5 mL were defined as having significant IMR. IMR was analyzed as a binary variable (presence/absence using the 5 mL cut-off) and by total and relative volume.

**Results:**

IMR was present in 8 out of 33 patients (24.2%), with 4 out of 11 (36.4%) having mTICI 2C, and 4 out of 22 (18.2%) having mTICI 3. After adjustment for relevant variables, absolute and relative IMR volumes were associated with higher National Institutes of Health Stroke Scale (NIHSS) scores at 5 days (adjusted beta =0.50 [0.05, 0.96], *p* = 0.03) and at 24 h (adjusted beta = 0.11 [0.02, 0.19], *p* = 0.01). No independent associations were found between IMR and the 90-day modified Rankin Scale (mRS).

**Conclusion:**

IMR is present in one-quarter of patients and is associated with worse early neurological outcomes.

## Introduction

The no-reflow (NR) phenomenon (1) has been defined as the absence of microvascular filling after endovascular treatment (EVT). Initially identified in preclinical models of the nervous system, NR is attributed to the obstruction of arterioles and capillaries resulting from microthrombi, endothelial swelling, and pericyte contraction (2, 3). Early *in vivo* descriptions of NR highlighted the absence of capillary blush in selective angiograms distal to the occlusion site after EVT (4). While microthrombi and pericyte contraction are central to the NR phenomenon, other factors—such as circulatory failure or vasogenic edema—may also play a role beyond its primary scope (5). To address these broader mechanisms, the term *impaired microvascular reperfusion* (IMR) *despite complete recanalization* is currently preferred. Unlike the NR concept, IMR offers a more pragmatic framework, including all forms of persistent hypoperfusion, regardless of the underlying cause.

The earlier studies on IMR utilized digital subtraction angiography (DSA), which was performed immediately after EVT. Despite the lack of validation in preclinical models (5), perfusion imaging techniques have become the preferred method for identifying IMR, relying on persistent hypoperfusion as a surrogate marker (6). The perfusion maps used, the inclusion criteria applied, and the definition of IMR on these maps have all varied. Some studies have focused on persistent hypoperfusion in relative cerebral blood volume (rCBV) or relative cerebral blood flow rCBF maps within the infarcted area (7), while others have evaluated it as persistent regions of Tmax>6 s outside or within the infarcted tissue (8). This variability across studies helps to explain the differences in reported prevalence (0–42.9%) and its impact on functional outcomes (7–9). Recently, ter Schiphorst’s review (5) proposed a set of inclusion criteria to establish a baseline quality standard.

This study aimed to investigate the prevalence and prognostic significance of impaired microvascular perfusion in a sample of patients achieving successful angiographic recanalization after acute ischemic stroke. We hypothesized that brain perfusion abnormalities after successful EVT are common and contribute to adverse clinical outcomes in stroke patients.

## Materials and methods

This study is a part of the prospective project Futile Reperfusion in Ischemic Acute Stroke (FURIAS). The clinical and radiological protocol of the FURIAS project was detailed in a previous study (10). The comparison of the characteristics of the eligible cohort can be found in Supplementary Table 2. The Research Ethics Committee of the Germans Trias I Pujol Hospital approved the study. All the patients or their relatives provided written informed consent. We recruited consecutive patients with anterior large vessel occlusion who underwent EVT. In this group of patients, we performed MRI at three time points: at hospital arrival (MRI pre-EVT), less than 2 h after endovascular treatment (MRI post-EVT), and 5 days after the stroke (MRI on day 5). All patients had an mRS score of less than 2 before the stroke and an NIHSS score of ≥6 upon admission. Additionally, they had time from onset to admission of ≤6 h, until the publication of the DAWN trial (11). After January 2018, we recruited patients with the DAWN criteria (11).

Inclusion criteria: For this sub-study, we included patients achieving final mTICI2c or mTICI3, with adequate perfusion imaging at arrival and post-EVT. Exclusion criteria: we excluded patients with an extracranial internal carotid artery (ICA) occlusion and those who presented a hemorrhagic transformation (parenchymal hematoma; PH or hemorrhagic infarct; IH) or an arterial reocclusion on the post-EVT MRI. Patients with ICA occlusion, patients with mTICI <2C, and patients presenting reocclusion in the magnetic resonance angiogram (MRA) post-EVT were excluded because some perfusion deficit is expected in such situations. Furthermore, patients showing any hemorrhagic transformation in the post-EVT MRI were excluded because blood can produce artifacts in the perfusion sequences.

### MRI protocol

All images were performed using a 3 Tesla Siemens Magnetom Verio (Siemens, Erlangen, Germany), except for nine MRIs at 5 days that were acquired on a 1.5 Tesla Philips (Philips Healthcare, Best, Netherlands). The MRI protocol included diffusion-weighted imaging (DWI), susceptibility-weighted imaging, fluid-attenuated inversion recovery (FLAIR), Time of flight MRA, and perfusion-weighted imaging (PWI). PWI was not performed at 5 days. Further information about the imaging protocol can be found elsewhere (10).

### Image analysis and post-processing

All the images were securely stored and pseudonymized for analysis. PWI images pre- and post-EVT were processed using Olea Sphere 3.0 – SP22 software (Olea Medical, La Ciotat, France) to obtain rCBV, rCBF, Tmax, and time-to-peak maps. FMRIB’s Linear Image Registration Tool (FLIRT) was used to perform a 6-degree corregistration of the post-EVT DWI (including the segmentations above) to the PWI post-EVT maps. We used mutual information as the evaluation metric during the registration process, and all the registrations were reviewed by an expert in neuroimaging (MH).

The pre-EVT MRI was utilized to assess penumbra volumes, while the post-EVT MRI enabled the application of the definition for IMR. Additionally, the 5-day MRI was used to evaluate outcomes, including final infarct volume and hemorrhagic transformation.

A stroke neurologist expert in neuroimaging (MH) manually segmented hyperintensities on DWI pre-EVT, post-EVT, and at day 5, creating lesion masks (12). Infarct volume (mm3) at each time point was calculated from the DWI masks, and hemorrhagic transformation was assessed on the MRI at 5 days according to the European Cooperative Acute Stroke Study II (ECASS-II) classification.

We obtained pre-EVT Tmax>6 s and Tmax>10s masks by applying the corresponding thresholds (6 and 10 s) to the pre-EVT Tmax maps. Hypoperfusion intensity ratio (HIR) was calculated on the pre-EVT maps by dividing the volume of tissue with Tmax >10s by the Tmax>6 s volume.

An experienced interventional neuroradiologist (MW) blinded to the clinical and radiological data evaluated all the angiographies and recorded the final arterial revascularization status according to the mTICI scale with 2c grades (12).

### Impaired microvascular reperfusion definition

**Figure 1:**
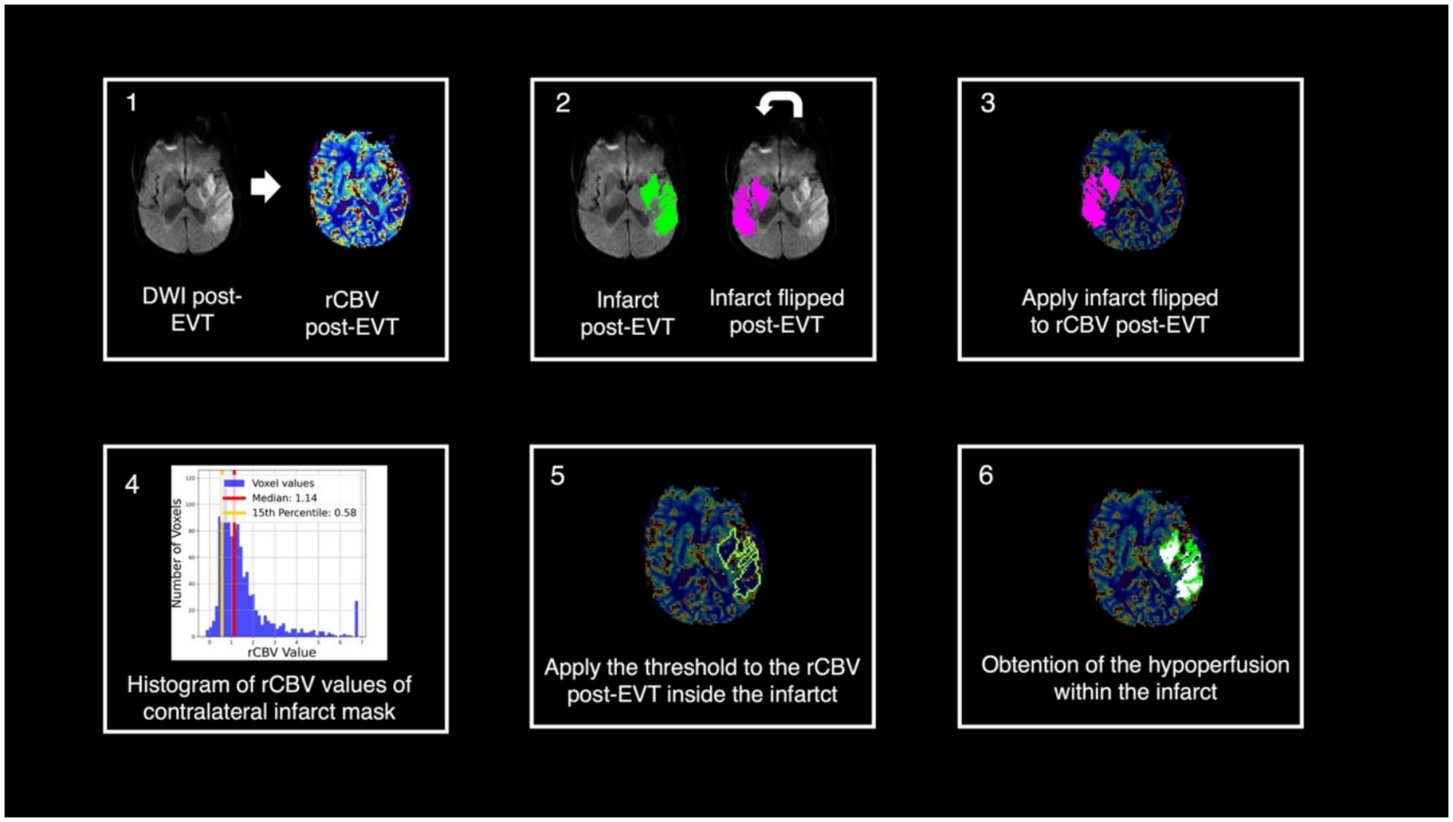
Pipeline of the post-processing. (1) Rigid registration of the DWI post-EVT and its infarct segmentation to the rCBV post-EVT. (2) Post-EVT Infarct flip. (3 and 4) Obtention of the voxel values of the rCBV post-EVT in the contralateral flipped infarct and determination of the 15% threshold. (5) Threshold application within the infarct segmentation. (6) Obtention of the infarct microvascular impairment segmentation. DWI, diffusion-weighted image; EVT, endovascular treatment, rCBV, relative cerebral blood volume.

Figure 1 shows the pipeline of the imaging post-processing. We obtained a specular mask of the post-EVT infarct volume in the contralateral hemisphere and calculated the median value of rCBV excluding cerebrospinal fluid. Voxels within the post-EVT infarct volume that exhibited at least a 15% reduction in the rCBV value relative to the mirrored mask volume’s median value were considered to have post-EVT hypoperfusion (7). Only clusters of more than 10 contiguous voxels with hypoperfusion were considered significant (5). All the final masks were reviewed by a vascular neurologist to discard artifacts and ensure the quality of the masks. We calculated the total volume of IMR and the median value of rCBV within IMR. Significant IMR was defined when a patient had an IMR volume beyond 5 mL. This cut-off was established based on previous literature, which suggested establishing a significant threshold (5) to evaluate perfusion deficits. All the images and masks were visually reviewed to exclude potential sequelae lesions associated with hypoperfusion.

Despite the comparability of rCBV and CBF maps in terms of IMR (13), rCBV was preferred due to its consistency in our cohort and its stronger association with capillary density and microvascular integrity, as demonstrated in other neurological conditions such as gliomas (14).

### Statistical analysis

The study described variables by mean (standard deviation), median (interquartile range, IQR), or absolute frequencies and percentages as appropriate. We used a paired Wilcoxon signed-rank test to compare the rCBV values in regions of interest with the contralateral side. Baseline characteristics were compared between patients with and without IMR using the chi-square, Fisher exact, or Mann–Whitney test. A Spearman’s rank correlation was conducted when evaluating associations between two quantitative variables.

We conducted multiple regression analyses to study the association between IMR (evaluated as a binary variable and as volumes) and clinical and radiological outcomes. Variables and models employed are found in Supplementary Table 1.

Statistical analysis was performed with R (22), with two-sided *p*-values <0.05 considered statistically significant.

All data are available upon reasonable request.

## Results

**Figure 2:**
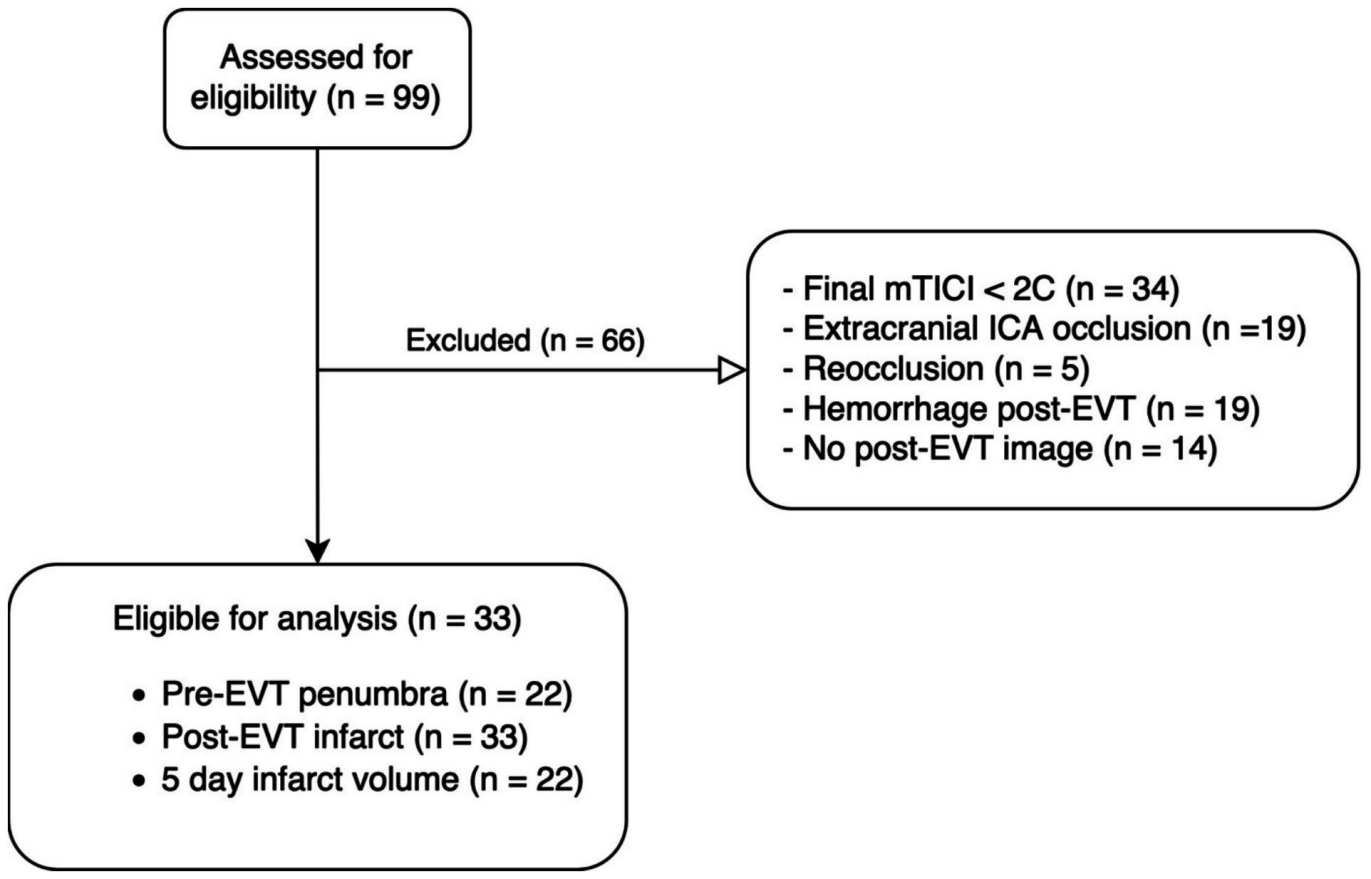
Consort diagram. EVT, Endovascular treatment; ICA, intracranial carotid artery; mTICI, modified Thrombolysis in Cerebral Infarction.

Between April 2015 and October 2018, we recruited 99 patients, out of which 33 patients were selected for analysis. Figure 2 reveals reasons for exclusion. The median (IQR) volume of infarct post-EVT and saved penumbra were 15.8 [6.23; 20.1] ml and 99.4 [50.7; 191], respectively. Infarcted and saved penumbra areas in post-EVT exhibited similar median rCBV values to their specular ROIs in the healthy hemisphere. Specifically, the infarcted region had an rCBV value of 1.54 [1.19–2.31] mL/100 g compared to 1.34 [0.96–1.79] mL/100 g for its specular ROI, *p* = 0.147. Similarly, the saved penumbra region had an rCBV value of 1.61 [1.10–2.05] mL/100 g, compared to 1.37 [1.13–2.00] mL/100 g for its specular ROI, with a *p*-value of 0.84.

### IMR and perfusion values within the infarct

**Figure 3:**
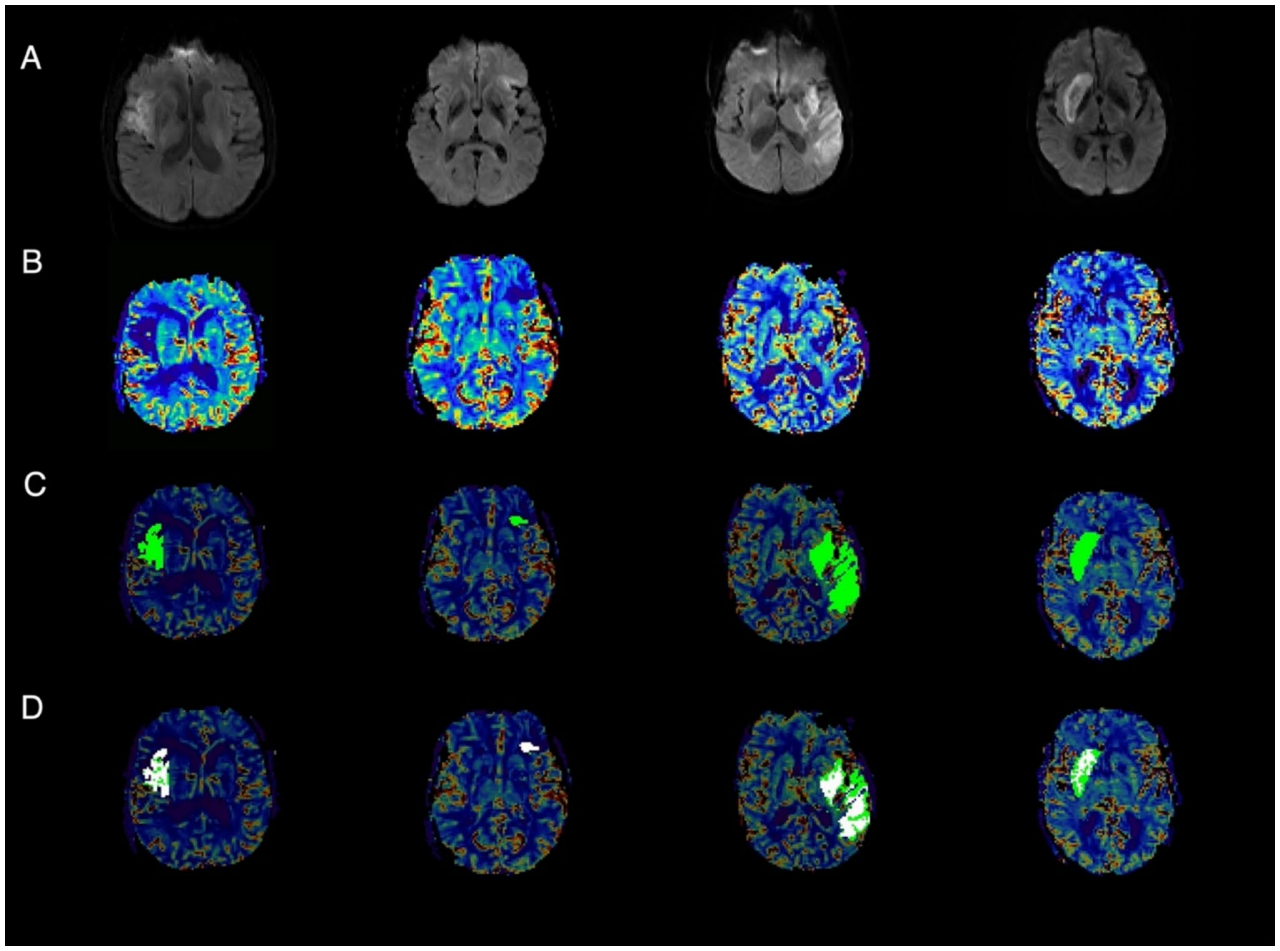
Representative cases of infarct microvascular impairment (IMR). **(A)** Post-EVT diffusion-weighted imaging (DWI); **(B)** Post-EVT relative cerebral blood volume (rCBV); **(C)** Post-EVT rCBV with overlaid infarct area (light green); **(D)** Post-EVT rCBV showing the infarct area (green) and regions of IMR (white).

Out of 33 patients analyzed, only 8 displayed significant IMR areas, which accounted for 24.2% of the patients. Figure 3 displays some representative cases. A significant IMR was present in 4 out of 11 (36.4%) patients achieving mTICI2c and in 4 out of 22 (18.2%) achieving mTICI3 (*p* = 0.39). In the patients with significant IMR, the median (IQR) IMR volume was 14.5 [11.4; 21.5] mL, with an area that represented 43 [34; 54] % of the infarcted tissue. Moreover, the IMR voxels had a median rCBV value of 0.58 [0.13; 0.86] mL/100 g.

Table 1 summarizes the baseline characteristics of the cohort and patients with significant IMR. The presence of significant IMR was associated with larger pre-EVT DWI lesions. In the adjusted analysis, the presence of significant IMR was not found to be associated with any outcome (Table 2).

**Table 1:** Baseline characteristics of the patients.

	All *N* = 33	No significant IMR *N* = 25	Significant IMR patients *N* = 8	*p*-value
Age (years), (median, IQR)	72.0 [69.0; 78.0]	72.0 [68.0; 81.0]	71.5 [70.8; 75.0]	0.659
Female sex (*n*, %)	17 (51.5%)	13 (52.0%)	4 (50.0%)	1.000
NIHSS score at admission (median IQR)	17.0 [12.0; 22.0]	16.0 [12.0; 21.0]	21.0 [16.8; 22.0]	0.302
Glycemia (mg/dl), (median IQR)	110 [99.5; 132]	110 [99.0; 130]	130 [104; 141]	0.372
Tmax>6 s volume at admission (ml), (median IQR)	103 [67.0; 140]	96.0 [46.0; 137]	126 [98.2; 214]	0.153
Saved penumbra volume (ml), (median IQR)	99.4 [50.7; 191]	155 [50.7; 201]	77.6 [54.5; 92.4]	0.268
Pre EVT infarct volume (ml), (median IQR)	10.1 [6.70; 15.7]	6.87 [6.05; 12.8]	35.9 [15.8; 61.5]	0.001
HIR (mean, SD)	0.41 [0.28; 0.55]	0.41 [0.24; 0.53]	0.48 [0.38; 0.72]	0.180
Time to treatment				1
<6 h	23 (69.7%)	17 (68.0%)	6 (75.0%)	
6-24 h	10 (30.3%)	8 (32.0%)	2 (25.0%)	
Time from symptom-onset-to-angiographic-reperfusion (minutes), (median IQR)	365 [255; 547]	335 [255; 565]	378 [287; 502]	0.900
Time to image (minutes), (median IQR)	270 [165; 448]	270 [165; 448]	273 [206; 379]	0.785
Treatment (*n*, %)				0.699
rTPA + EVT	15 (45.5%)	12 (48.0%)	3 (37.5%)	
Primary EVT	18 (54.5%)	13 (52.0%)	5 (62.5%)	
Final mTICI *n* (*n*, %)				0.391
mTICI 2c	11 (33.3%)	7 (28.0%)	4 (50.0%)	
mTICI 3	22 (66.7%)	18 (72.0%)	4 (50.0%)	

IMR, impaired microvascular reperfusion; HIR, Hypoperfusion intensity ratio; IQR, interquartile range; SD, standard deviation; rTPA, recombinant tissue plasminogen activator; EVT, endovascular treatment; mTICI, modified Thrombolysis in cerebral infarction; NIHSS, National Institute of Health Stroke Scale.

**Table 2:** Multiple regression analysis of IMR.

	Significant IMR	IMR volume	% of IMR volume within the infarct
NIHSS score at 24 h (*β*, 95% CI)	5.03 [−3.04, 13.10], *p* = 0.21	0.15 [−0.07, 0.36], *p* = 0.17	0.11 [0.02, 0.19], *p* = 0.01
NIHSS score 5 days or discharge (*β*, 95% CI)	6.81 [−4.79, 18.41], *p* = 0.24	0.50 [0.05, 0.96], *p* = 0.03	0.12 [−0.05, 0.28], *p* = 0.17
mRS score at 3 months (aOR, 95% CI)	0.72 [0.08, 5.97], *p* = 0.77	1.02 [0.90, 1.14], *p* = 0.76	1.00 [0.97, 1.04], *p* = 0.92
Functional independence (acOR, 95% CI)	1.30 [0.02, 68.19], *p* = 0.89	1.11 [0.78, 1.55], *p* = 0.55	1.02 [0.94, 1.11], *p* = 0.58
Any hemorrhagic transformation (aOR, 95% CI)	2.12 [0.42, 12.34], *p* = 0.37	1.05 [0.99, 1.19], *p* = 0.29	1.00 [0.96, 1.03], *p* = 0.91
Growth >10 mL at 5 days (aOR, 95% CI)	2.03 [0.11, 77.79], *p* = 0.65	1.05 [0.84, 1.36], *p* = 0.69	1.01 [0.96, 1.09], *p* = 0.62
Infarct volume at 5 days (*β*, 95% CI)	1.91 [−24.63, 28.45], *p* = 0.88	0.15 [−2.64, 2.94], *p* = 0.91	0.03 [−0.42, 0.48], *p* = 0.90

Betas of the covariates can be found in Supplementary Table 3.

IMR, impaired microvascular reperfusion; EVT, endovascular treatment; aOR, adjusted odds ratio; acOR, adjusted common odds ratio; CI, confidence interval; mRS, modified Rankin scale; NIHSS, National Institutes of Health Stroke Scale.

When evaluated quantitatively, IMR volume was associated with post-EVT infarct volume (rho = 0.82, *p* < 0.01), infarct volume at day 5 (rho = 0.63, *p* < 0.01), NIHSS scores at 24 h (rho = 0.57, *p* < 0.01) and 5 days (rho = 0.63, *p* < 0.01), and mRS scores at 90 days (common OR = 1.09 [1.01; 1.18], *p* = 0.03). In the adjusted analysis, absolute and relative IMR volume remained significantly associated with the NIHSS scores at 5 days and 24 h, respectively (Table 2).

Post-EVT rCBV values within the infarct were not linked with final mTICI, NIHSS, hemorrhagic transformation, infarct growth, use of rTPA, or functional outcome at 3 months. The IMR frequency was similar in rTPA-treated and untreated patients (5/18 (27.8%) and 3/15 (20%) *p* = 0.699, respectively).

## Discussion

Our data reveal that significant IMR is present in approximately one-quarter of patients following successful EVT. While a larger IMR volume was initially associated with a worse prognosis, this relationship did not persist after adjustment for confounding factors.

The prevalence of persistent perfusion deficits is debated; our study reported a 24.2% occurrence (18.2% in patients with final mTICI 3), contrasting with other rates ranging from 0 to 42.5% (6, 7, 15, 16). Discrepancies may arise from different imaging techniques, perfusion thresholds, timing of imaging, and inclusion criteria. Using definitions and exclusion criteria similar to previous studies (7), IMR rates in our cohort were comparable (25–29%) despite earlier imaging after recanalization.

Studies performing imaging within 30 min after EVT showed a delay exceeding Tmax >6 s in 42.5% of mTICI3 patients (6). However, these studies applied less stringent criteria, including patients with ICA occlusions (17% had tandem lesions) and post-EVT hematomas, potentially inflating IMR prevalence. In contrast, other studies using a more similar methodology than ours (7), but without excluding hemorrhagic areas, reported IMR in 25.3% of patients when imaging was acquired up to 24 h after complete recanalization. When researchers included mTICI 2c-3 patients and excluded the three possible causes of apparent persistent hypoperfusion (ICA stenosis, intracranial reocclusion, or areas of hemorrhage), the prevalence of IMR was dramatically reduced to 3.33% (16) or 0% (17).

Compared to those with similar selection criteria, our higher prevalence may be attributed to the shorter imaging acquisition time (<2 h vs. up to 24 h), the longer onset-to-recanalization time ([255–547 min] vs. [61–367 min]), and the use of a less stringent method for assessing hypoperfusion (15% reduction compared to the mirror region vs. 40% of the mirror region or visual assessment). Recent research (13) has explored the optimal methodology for evaluating IMR by comparing four different definitions (6, 7, 16, 18) in a common cohort of 131 patients. Although the reported prevalence of IMR varied widely (0.8–22.1%), the definition employed in our study (7) proved to be the most effective in predicting functional outcomes at 3 months. While we assumed a 5-mL volume threshold (5) to determine significant IMR, it has not been validated, highlighting the need to establish a volume cut-off to determine relevant IMR. Additionally, the 15% difference compared to the mirror region is supported by limited evidence (side-to-side variations of 14.5% on SPECT between healthy hemispheres are considered normal (19, 20)), emphasizing the necessity for thresholds derived from preclinical studies that also account for distinctions between white and gray matter.

Consistent with previous literature (7), we found that IMR volume was associated with higher NIHSS scores at 5 days (absolute) and 24 h (relative). Still, we could not find any significant association between other outcomes and IMR in its current definition. Other studies have shown that persistent Tmax>6 s, either inside or outside the infarct, is associated with perfusion derangement and functional outcomes (8, 18), and in a previous study of our cohort, including patients with any final mTICI, post-EVT Tmax>6 s volume was associated with infarct growth (10).

Our analysis was possibly underpowered due to the small sample size; thus, the lack of association of IMR with outcomes could be a type II error. Given the stringent exclusion criteria (with 66% of our sample being excluded) and the intrinsic complexity of conducting repeated image studies with stroke patients shortly after revascularization, overcoming this obstacle will require the implementation of a well-designed multicentric study. While the prevalence of IMR is lower than what is typically observed in clinical practice after excluding all potential causes, it is still more common than earlier studies have suggested (16, 17). The strict selection criteria inherently limit the sample size [*n* = 27 and *n* = 33 in (16, 17)], making it challenging to identify statistically significant associations. Therefore, we call for the collaboration of other centers to continue this line of research.

Our study offers several strengths. We exclusively used MRI perfusion imaging, yielding unique data on perfusion values post-EVT. Unlike previous studies (6, 7, 9) that incorporate both CT and MRI, this approach helps reduce variability. In addition, our computational approach provides precise quantification of perfusion abnormalities and infarct characteristics, minimizing subjectivity and enhancing reproducibility compared to traditional visual assessment methods. Compared to other methods that consider the whole infarct as either affected or unaffected, our approach permits the segmentation of IMR, delimiting its extent to certain areas of the infarct, which allows its quantification.

However, like other methods, this approach struggles to differentiate distinct IMR thresholds between gray and white matter. Given the lower rCBV levels in white matter, particularly in infarcts affecting both gray and white matter, our method may be less sensitive in segmenting IMR voxels in the gray matter and less specific in identifying affected voxels in the white matter. In such cases, determining a separate threshold for each tissue type would be necessary. However, due to the disruption of tissue integrity following an infarct, applying such thresholds in the infarcted area would likely be challenging.

Additionally, we defined infarct based on DWI volume within 2 h after EVT, potentially underestimating the accurate infarct volume due to partial DWI’s reversibility. Despite this, we found no differences in the presence of IMR among mTICI2c and mTICI3 grades. While we did not apply *p*-value corrections due to ongoing debates on their utility (21), our findings align with existing literature.

Our results emphasize the prognostic importance of post-EVT perfusion yet highlight the need for a consistent IMR definition. The dynamic nature of cerebral perfusion after reperfusion therapies complicates this effort, requiring consideration of imaging timing and thresholds. Radiological definitions should align with anatomopathological criteria, necessitating further radiological-pathological studies to establish a unified IMR concept, identify contributing factors, and develop therapeutic strategies.

In summary, IMR volume is significantly and independently associated with poor outcomes in ischemic stroke patients. However, additional research is required to validate these findings and investigate optimal timing and definitions of IMR.

## Data Availability

The raw data supporting the conclusions of this article will be made available by the authors, without undue reservation.

## Author’s note

This article was presented as a poster at the ESOC (European Stroke Organization Conference) 2023.
