# Supplementary material for: Relevance of persistent perfusion deficits on clinical outcomes after successful endovascular treatment: a prospective serial magnetic resonance study
Source: Front Neurol. 2025 Feb 27;16:1478240. doi: 10.3389/fneur.2025.1478240 (PMC11905896; doi:10.3389/fneur.2025.1478240)
Supplement: Supplementary file 1 [file Table_1.docx]

Supplementary table 1: Models and covariates

| Outcome | Model | Covariates for analysis |
| --- | --- | --- |
| NIHSS at 24 h | Median regression | NIHSS at admission, age |
| NIHSS at 5 days | Median regression | NIHSS at admission, age |
| mRS at 3 months | Ordered logistic regression | NIHSS at admission, age, post-EVT infarct volume |
| Hemorrhagic transformation at 5 days | Binomial logistic regression | Age, rTPA treatment |
| Infarct volume at 5 days | Median regression | Age, post-EVT infarct volume, duration of ischemia |
| Growth > 10 ml at 5 days | Binomial logistic regression | Age, post-EVT infarct volume, duration of ischemia |

EVT = Endovascular treatment; mRS = modified Rankin scale. rTPA = recombinant tissue plasminogen activator

|  | Screened (n=99) | Excluded (n=224) |
| --- | --- | --- |
| Age | 70±13 | 70±12 |
| Sex (male) | 57 (57.6%) | 124 (55.4%) |
| Site of occlusion |  |  |
| Tandem | 23 (23.2%) | 34 (15%) |
| TICA | 10 (10.1%) | 44 (19%) |
| M1 | 52 (52.5%) | 118 (53%) |
| M2 | 14 (14.1%) | 28 (13%) |
| NIHSS at baseline | 18 [12-21] | 18 [13-22] |
| Time onset to arrival (min) | 257 [137-445] | 207 [70-319] |

Supplementary table 2: characteristics of the eligible sample. The full cohort comes from the FURIAS study

| Outcome | Predictor | Covariates | | | | |
| --- | --- | --- | --- | --- | --- | --- |
|  |  | NIHSS at admission | Age (years) | post-EVT infarct volume (ml) | rTPA treatment (yes/no) | Duration of ischemia (min) |
| NIHSS at 24 h (β, 95% CI) | Significant IMR | 0.35 [0.09, 0.62], p=0.01 | -0.08 [-0.19, 0.03], p=0.17 |  |  |  |
|  | Absolute IMR volume | 0.42 [-0.08, 0.91], p=0.10 | 0.02 [-0.19, 0.22], p=0.85 |  |  |  |
|  | Relative IMR volume | 0.64 [0.13, 1.15], p=0.02 | 0.02 [-0.19, 0.22], p=0.86 |  |  |  |
| NIHSS 5 days or discharge (β, 95% CI) | Significant IMR | 0.38 [0.04, 0.71], p=0.03 | 0.07 [-0.08, 0.22], p=0.37 |  |  |  |
|  | Absolute IMR volume | 0.29 [-0.20, 0.78], p=0.23 | 0.06 [-0.08, 0.21], p=0.40 |  |  |  |
|  | Relative IMR volume | 0.51 [0.13, 0.89], p=<0.01 | 0.03 [-0.11, 0.18], p=0.62 |  |  |  |
| mRS score at 3 months (aOR, 95% CI) | Significant IMR | 1.07 [0.94, 1.23], p=0.33 | 1.04 [0.98, 1.11], p=0.22 | 1.06 [1.02, 1.12], p=0.04 |  |  |
|  | Absolute IMR volume | 1.08 [0.95, 1.24], p=0.28 | 1.04 [0.97, 1.11], p=0.25 | 1.05 [1.01, 1.11], p=0.03 |  |  |
|  | Relative IMR volume | 1.07 [0.94, 1.24], p=0.30 | 1.04 [0.98, 1.11], p=0.23 | 1.05 [1.02, 1.10], p=0.02 |  |  |
| Functional independence (acOR, 95% CI) | Significant IMR | 1.17 [0.91, 1.60], p=0.25 | 1.31 [1.08, 1.81], p=0.03 | 1.07 [1.00, 1.26], p=0.36 |  |  |
|  | Absolute IMR volume | 1.20 [0.92, 1.63], p=0.19 | 1.31 [1.08, 1.86], p=0.04 | 1.03 [1.00, 1.26], p=0.69 |  |  |
|  | Relative IMR volume | 1.19 [0.92, 1.64], p=0.22 | 1.34 [1.09, 2.03], p=0.05 | 1.05 [1.00, 1.24], p=0.43 |  |  |
| Any haemorrhagic transformation (aOR, 95% CI) | Significant IMR |  | 1.01 [0.94, 1.08], p=0.79 |  | 1.14 [0.26, 4.98], p=0.86 |  |
|  | Absolute IMR volume |  | 1.01 [0.94, 1.09], p=0.75 |  | 1.20 [0.27, 5.51], p=0.81 |  |
|  | Relative IMR volume |  | 1.01 [0.94, 1.08], p=0.82 |  | 1.19 [0.28, 5.12], p=0.81 |  |
| Growth > 10 ml at 5 days (aOR, 95% CI) | Significant IMR |  | 1.08 [0.98, 1.23], p=0.15 | 0.99 [0.94, 1.05], p=0.84 |  | 1.00 [1.00, 1.01], p=0.32 |
|  | Absolute IMR volume |  | 1.08 [0.99, 1.23], p=0.14 | 0.99 [0.93, 1.05], p=0.83 |  | 1.00 [1.00, 1.01], p=0.31 |
|  | Relative IMR volume |  | 1.08 [0.99, 1.23], p=0.13 | 1.00 [0.96, 1.05], p=0.94 |  | 1.00 [1.00, 1.01], p=0.39 |
| Infarct volume at 5 days (β, 95% CI) | Significant IMR |  | 0.39 [-0.20, 0.99], p=0.18 | 1.26 [0.98, 1.54], p=<0.01 |  | 0.01 [-0.01, 0.04], p=0.29 |
|  | Absolute IMR volume |  | 0.39 [0.10, 0.68], p=0.01 | 1.25 [1.04, 1.45], p=<0.01 |  | 0.01 [0.00, 0.03], p=0.14 |
|  | Relative IMR volume |  | 0.33 [0.13, 0.52], p=<0.01 | 1.22 [0.86, 1.58], p=<0.01 |  | 0.01 [0.00, 0.02], p=0.12 |

Supplementary table 3: Adjusted betas and covariates of the models. aOR=adjusted odds ratio; acOR=adjusted common odds ratio, IMI=infarct microvascular impairment; EVT = endovascular treatment; NIHSS = National Institute of Health Stroke Scale; rTPA= recombinant tissue plasminogen activator,
